# The Association Between Maternal Short Stature and Neonatal Intensive Care Unit Admission: A Longitudinal Study in Sabah

**DOI:** 10.7759/cureus.48924

**Published:** 2023-11-16

**Authors:** Ehab Helmy, Helen Benedict Lesimbang, M Tanveer Hossain Parash, Soon Ruey, Nurhidayah Binti Kamarudin, Ong Teck Siong, Teoh Jie Sheng, Khairul Sabrin Bin Ahmad, Syaza Nadia Binti Saman, Kueh Bing Ling

**Affiliations:** 1 Department of Obstetrics and Gynaecology, Faculty of Medicine and Health Sciences, Universiti Malaysia Sabah, Kota Kinabalu, MYS; 2 Anatomy Unit, Department of Biomedical Sciences, Faculty of Medicine and Health Sciences, Universiti Malaysia Sabah, Kota Kinabalu, MYS; 3 Department of Obstetrics and Gynaecology, Hospital Wanita Dan Kanak-Kanak Sabah, Kota Kinabalu, MYS; 4 Clinical Research Centre, Hospital Wanita Dan Kanak-Kanak Sabah, Kota Kinabalu, MYS

**Keywords:** malaysia, sabah, longitudinal studies, maternal short stature, low birth weight (lbw), nicu admissions

## Abstract

Background: The rising number of newborns requiring neonatal intensive care unit (NICU) care poses immediate threats to their health and places emotional and financial burdens on families and healthcare systems. This study investigates the direct effect of maternal short stature on NICU admission in Sabah, Malaysia.

Methods: A longitudinal study at Hospital Wanita Dan Kanak-Kanak Sabah (HWKKS) from 2018 to 2022 included 254 Malaysian women with singleton pregnancies and neonates born after the 37th week, excluding significant disorders, smoking/alcohol use, fetal death, and malformations. Birth weight, gestational age, and neonatal condition were recorded. The association between maternal height, low birth weight (LBW), and NICU admission was analyzed.

Results: LBW prevalence was 15.35%, with an average participant height of 147.37 cm. Maternal stature was significantly associated with LBW, with the shortest quartile (Q1) having the highest risk. LBW was significantly associated with NICU admission, with LBW newborns at a sixfold higher risk. Maternal height was also significantly associated with NICU admission, with Q1 having the highest risk. The receiver operating characteristic (ROC) curve suggested combining Q1 and Q2 for the best prediction of NICU admission, indicating that shorter mothers face a higher risk of neonates requiring NICU care.

Conclusion: Maternal short stature could be a valuable predictor of LBW and NICU admission risk. It may be a screening tool to assess these risks in healthcare settings. However, further research is needed to explore this association's underlying mechanisms and potential interventions.

## Introduction

Neonatal mortality has decreased across many regions globally owing to progress in perinatal medicine and the establishment of neonatal intensive care units (NICUs) [1-3]. Additionally, highly preterm infants' survival rates have improved from 76% in 2012 to 78.3% in 2018 [4-6]. Concurrently, there has been a rise in the number of newborns necessitating intensive care, with NICU admissions increasing from 6.4% in 2007 to 7.2% in 2018 [7,8]. NICU admission leads to an instant threat of morbidity and mortality for neonates. It also places a significant emotional and financial strain on their families and the healthcare system [9,10].

Inoue et al. (2016), at a general hospital in Japan, observed that low birth weight (LBW) newborns were more likely to experience NICU admission (12.1%) [11]. Haidari et al. (2021) noted that in contrast to infants who did not require NICU admission, those who were admitted exhibited a significantly higher likelihood of having lower birth weight, being born at an earlier gestational age, having mothers with lower parity, experiencing congenital malformations, or having a five-minute Apgar (appearance, pulse, grimace, activity, and respiration) score of less than 7 [12]. In Malaysia, the number of LBW infants admitted to NICUs increased from 32.8% to 38.6% between 2012 and 2016 [13]. In the multiple logistic regression model based on 10-year (2009-2018) data, Boo et al. (2021) found that LBW was one of the significant predictors of mortality among very preterm neonates in the Malaysian National Neonatal Registry [14].

Low birth weight poses a substantial concern for public health [15]. The World Health Organization characterizes LBW as a birth weight below 2,500 g, regardless of the infant's gestational age [16]. About 80% of annual neonatal deaths result from low birth weight, deriving from factors such as preterm birth, being small for their gestational age, or a combination of both [17,18]. Surviving LBW infants face an elevated risk of experiencing adverse health outcomes in the short and long term [19]. The causes of low birth weight are multifaceted, and maternal short stature plays a pivotal role with intergenerational implications [20]. Numerous studies investigating the association between maternal height and low birth weight have demonstrated that shorter maternal stature is linked to decreased fetal growth and a higher likelihood of LBW [11,21-23]. Therefore, it can be postulated that maternal short stature could increase the risk of LBW newborns requiring NICU admission. This study aims to keep maternal short stature as a direct risk factor for NICU admission at term pregnancy in Sabah, Malaysia. To our knowledge, no studies of the Sabah population have demonstrated the effect of shorter maternal height on newborns' health outcomes.

## Materials and methods

Study design

This longitudinal study was conducted at the Hospital Wanita Dan Kanak-Kanak Sabah (HWKKS) from September 1, 2018, to December 31, 2022.

Study population

Sample Size

The minimum sample size was calculated as 250 using the sample size calculator provided by the Australian Bureau of Statistics, where the confidence level is 95%, the proportion is 50%, the confidence interval (CI) is 0.05, the standard error is 0.02, and the relative standard error is 4.75. The population size is 568 [24,25].

Inclusion criteria

The study included Malaysians aged more than 18 years having singleton pregnancies who planned to deliver in HWKKS and live-born neonates born after the 37th gestational week to avoid confounding LBW with a mature birth and preterm birth.

Exclusion criteria

The exclusion criteria were mothers having any major medical disorder resulting in LBW babies, smoking and alcohol consumption habits for any of the parents, intrauterine fetal death, and neonates with major congenital malformations.

Study procedure

Initially, 400 pregnant women who provided written informed consent were followed up monthly from their 36th-week visit to HWKKS to record their data. The aim was to recruit all live-born singleton babies without major congenital malformations. Gestational age was derived from the last menstrual period (LMP) unless it differed from that derived from an early ultrasound scan (<20 weeks gestation) by more than two weeks, in which case the latter was used.

The participant's information was collected under general clinical practice and recorded in individual medical records. The data included age, smoking habits, alcohol drinking habits, newborns' gender, and the condition of the neonates after delivery.

**Figure 1:**
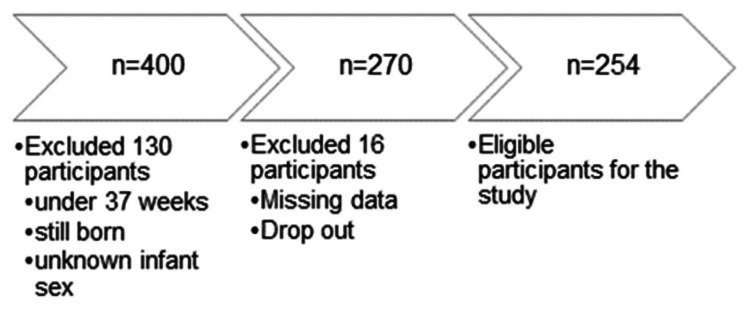
Participants' selection flow

We divided maternal height into four categories at quartile (Q) points, following Inoue et al. (2016), as Malaysian adult females had a similar average height (156.8±7 cm) with the Japanese population (157 cm): first quartile (Q1), 130-139.9 cm; second quartile (Q2), 140-144.9 cm; third quartile (Q3), 145-149.9 cm; and fourth quartile (Q4), 150-160 cm [11,25]. Maternal height was the exposure variable, and LBW and admission to the neonatal intensive care unit were the outcome variables. The mothers' heights of all live-born term singletons without major congenital malformations were recorded. A total of 254 participants fulfilled all the inclusion criteria (Figure 1).

Measurement of the babies

Birth weight was measured in the labor ward using a digital electronic weighing scale. A trained observer took measurements of head circumference, mid-upper arm circumference (MUAC), and abdominal circumference above umbilical cord insertion in expiration using blank paper tapes, marked and measured against a steel ruler.

Ethical statement

The Medical Research and Ethics Committee, Ministry of Health, Malaysia (NMRR ID: NMRR-18-2758-43077) gave ethical permission for the study.

Statistical analysis

A descriptive analysis was conducted to show the demographic characteristics of the mothers and newborns. The frequency of the demographic profile of the mothers and the neonates was expressed in percentages. The mean and standard deviations for the normally distributed data, and median and interquartile range (IQR) for the skewed distribution have been reported. We evaluated the relationships among maternal height, LBW, and NICU admission by chi-squared test. Relative risk (RR) and 95% confidence intervals (CIs) and probabilities estimated the risks of different quartiles of height of mothers having neonates being LBW and NICU admission. A p<0.05 was considered significant for this study. We used the Statistical Package for the Social Sciences (SPSS) version 22.0 for Windows (IBM SPSS Statistics, Armonk, NY) and SmartPLS 4 to conduct the statistical analyses in this study.

## Results

Most of the 254 participants were sure about their last menstrual period (LMP), but one-third were uncertain. One-fourth of the participants were primigravida, and the placenta was typically positioned in most cases (85%). Only a few (20%) had a bad obstetric history. Again, most newborns (65%) were delivered through expected vaginal delivery, and gender was almost equally distributed among them (50.79% females and 49.21% males). The prevalence of LBW was 15.35% (Table 1).

**Table 1:** Frequency distribution of the demographic profile of the participants (N=254) LMP: last menstrual period

		Low birth weight	
		No	Yes
Gravida	Primigravida	55 (83.3%)	11 (16.7%)
	Multigravida	160 (85.1%)	28 (14.9%)
Knowledge about LMP	Uncertain	74 (76.3%)	23 (23.7%)
	Certain	141 (89.8%)	16 (10.2%)
Bad obstetric history	Absent	173 (85.2%)	30 (14.8%)
	Present	42 (82.4%)	9 (17.6%)
Placental position	Placenta previa	6 (100%)	0 (0%)
	Fundus	26 (81.2%)	6 (18.8%)
	Anterior	91 (85%)	16 (15%)
	Posterior	92 (84.4%)	17 (15.6%)
Mode of delivery	Vaginal	139 (84.2%)	26 (15.8%)
	Cesarean section	76 (85.4%)	13 (14.6%)
Gender of neonate	Boy	104 (83.2%)	21 (16.8%)
	Girl	111 (86%)	18 (14%)
Total		215 (84.6%)	39 (15.4%)

The average height of the participants was 147.37 cm. The median weight of the neonates at the 28th week was 147.37 gm, and the mean weight at the 36th week and birth was 2,305.99 gm and 2,891.79 gm, respectively (Table 2).

**Table 2:** Descriptive statistics of the participants (N=254) EFW: estimated fetal weight

	Minimum	Maximum	Mean	Standard deviation	Median	Interquartile range
Maternal height (cm)	131	160	147.37	5.70	-	-
EFW at 28th week (gm)	631.6	3,789			1,188	219
EFW at 36th week (gm)	1,438	3,100	2,305.99	404.27		
Birth weight (gm)	1,630	3,860	2,891.79	400.19		

Out of 254 participants, 6.69% were less than 140 cm (Q1), 29.53% were between 140 and less than 145 cm (Q2), 31.50% were between 145 and less than 150 cm, and the rest (32.28%) were more than 150 cm (Q4) in height. There was a statistically significant (p<0.001) association between the different quartiles of the participants' stature and the low birth weight of the babies (Table 3).

**Table 3:** Association between the stature of the mother and the low birth weight of the newborn (N=254) Q1: first quartile, Q2: second quartile, Q3: third quartile, Q4: fourth quartile

	Low birth weight		Total	Chi-square statistic	Df	p-value
	No	Yes				
Q1 (130-139.9 cm)	9 (52.9%)	8 (47.1%)	17 (100%)	19.195	3	<0.001
Q2 (140-144.9 cm)	61 (81.3%)	14 (18.7%)	75 (100%)			
Q3 (145-149.9 cm)	68 (85%)	12 (15%)	80 (100%)			
Q4 (150-160 cm)	77 (93.9%)	5 (6.1%)	82 (100%)			
Total	215 (84.6%)	39 (15.4%)	254 (100%)			

From Table 4, if Q1 participants were pregnant, they were 3.6 times more likely to have low birth weight newborns. The expected risks of LBW newborns for the Q2 and Q3 were 1.3 and 0.97, respectively. The Q1 participants exhibited the highest risks. If a participant was less than 140 cm in height, she had the risk of having approximately 260% more LBW than others. In the case of Q2, the risk was 30%, and in Q3, the risk was only 4%, and it was not statistically significant (p>0.05).

**Table 4:** Risks of LBW for different quartiles of maternal stature (N=254) CI: confidence interval, LBW: low birth weight, Q1: first quartile, Q2: second quartile, Q3: third quartile, Q4: fourth quartile

Maternal stature			LBW		Relative risk	95%CI		z-statistic	p-value
			No	Yes		Lower	Upper		
Q1	No		206	31	3.598	1.971	6.566	4.171	<0.0001
	Yes		9	8					
Q2	No		154	25	1.337	0.736	2.426	0.954	0.340
	Yes		61	14					
Q3	No		147	27	0.967	0.517	1.808	0.106	0.916
	Yes		68	12					
Q4		No	138	34	0.309	0.125	0.760	2.558	0.011
		Yes	77	5					
Total			215	39	-	-	-	-	-

When the association between LBW and NICU admission was assessed, there was a significant association between neonate's LBW and ICU admission. The risk was six times higher than normal-weight newborns (Table 5).

**Table 5:** Association of LBW with NICU admission (N=254) CI: confidence interval, LBW: low birth weight, NICU: neonatal intensive care unit

LBW	NICU admission		Relative risk	95%CI		z-statistic	p-value
	No	Yes		Lower	Upper		
No	208	7	6.300	2.424	16.378	3.776	<0.001
Yes	31	8					
Total	239	15	-	-	-	-	-

Then, the relationship between maternal stature and NICU admission was examined. There was a statistically significant association between Q1 and NICU admission and Q2 and NICU admission. The risk of a Q1 mother having a newborn requiring NICU admission was 7.4 times more than that of non-Q1 mothers, whereas the risk for Q2 mothers was 2.3 (Table 6).

**Table 6:** Short stature of mother influencing children's NICU admission (N=254) CI: confidence interval, NICU: neonatal intensive care unit, Q1: first quartile, Q2: second quartile, Q3: third quartile

Maternal stature		NICU admission		Relative risk	95%CI		z-statistic	p-value
		No	Yes		Lower	Upper		
Q1	No	228	10	7.438	2.886	19.168	4.154	<0.0001
	Yes	11	5					
Q2	No	172	5	2.364	1.026	5.448	2.019	0.044
	Yes	67	10					
Q3	No	157	15	0.067	0.004	1.110	1.887	0.059
	Yes	82	0					
Total		239	15	-	-	-	-	-

Among the indications for newborns to be admitted to the NICU, transient tachypnea was the most common, followed by neonatal jaundice and glucose-6-phosphate dehydrogenase (G6PD) deficiency. Only one neonate was acknowledged for very low birth weight (<1,600 gm) (Table 7).

**Table 7:** Indication for NICU admission (n=15) G6PD: glucose-6-phosphate dehydrogenase, GDM: gestational diabetes mellitus, NICU: neonatal intensive care unit, RDS: respiratory distress syndrome, TTN: transient tachypnea of the newborn

Indication for NICU admission	Frequency	Percent
TTN	5	33.33
Neonatal jaundice	3	20
G6PD deficiency	2	13.32
Very low birth weight	1	6.67
Hypoglycemia	1	6.67
Infant GDM	1	6.67
Presumed sepsis	1	6.67
RDS prematurity	1	6.67
Total	15	100

**Figure 2:**
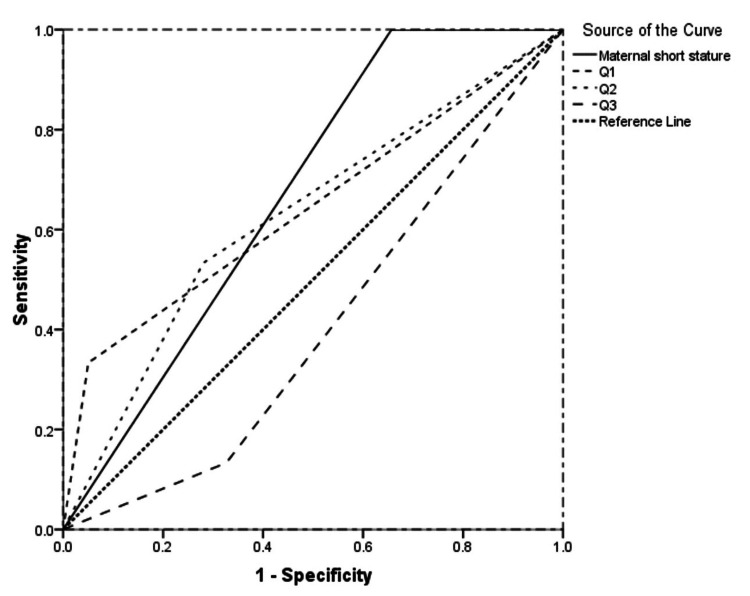
ROC curve for Q1, Q2, Q3, and short stature (Q1 and Q2 together) ROC: receiver operating characteristic, Q1: first quartile, Q2: second quartile, Q3: third quartile

When tested for how effective maternal stature is in predicting NICU admission, the receiver operating characteristic (ROC) curve (Figure 2) yielded that if maternal short lengths (Q1 and Q2) were combined, it could indicate the best, followed by Q1 and Q2 alone (Table 8). The area under the curves (AUCs), except for the model Q3, were satisfactory [26].

**Table 8:** AUC AUC: area under the curve, CI: confidence interval, Q1: first quartile, Q2: second quartile, Q3: third quartile

Test result variable(s)	AUC	Standard error	95%CI	
			Lower bound	Upper bound
Maternal short stature	0.672	0.054	0.566	0.777
Q1	0.642	0.085	0.474	0.809
Q2	0.626	0.078	0.474	0.779
Q3	0.403	0.068	0.270	0.537

## Discussion

The average height of the participants of this study was less than that of females reported by Chia et al. (2023) [25]. As most of the participants belonged to the short-stature group, the average height of the participants was well below the population's average height. The present study has identified that among mothers who gave birth at full term (beyond the 37th gestational week), shorter maternal stature was linked to the occurrence of LBW, with this association being more pronounced among mothers in the shortest height quartile (Q1), which corresponds to Inoue et al. (2016) [11]. This finding aligns with a study by Witter and Luke (1991) conducted in the United States, which reported that shorter women are more likely to have smaller newborns than taller women [21], corroborating our study's results. Additionally, Wills et al. (2010) demonstrated a relationship between parental height and fetal growth in the United Kingdom. However, they did not observe a clear association between maternal height and lower birth weight [22]. Notably, nutritional status is similar in these countries, with stable food supplies at the national level.

Consequently, shorter height may not indicate undernutrition; hereditary factors or other environmental variables likely influence it [11]. Observational epidemiological studies show that maternal height is associated with gestational age and fetal growth measures (i.e., shorter mothers deliver infants at earlier gestational ages with lower birth weight and birth length) [11,21-23,27,28]. Different mechanisms have been suggested to explain these associations [27]. Recent studies have employed anthropometric measurements, including height and weight, to predict poorer birth outcomes [11].

Women with shorter stature face an increased, unadjusted risk of experiencing preterm labor and giving birth to low birth weight (LBW) infants. Conversely, taller women exhibit roughly half the risk of LBW compared to average height [29]. The reduced height of some mothers may be attributed to a narrower pelvis, which can limit the available intrauterine space and potentially restrict fetal growth [30-32]. These differences in pelvic size are influenced by individual body size and can be expected in various settings. However, this alone does not comprehensively explain the relationship between maternal height and LBW. Other factors may contribute to the restricted growth of newborns. Multiple studies investigating the connection between maternal height and low birth weight have found that shorter maternal stature is linked to diminished fetal growth and LBW. They have concluded that the primary underlying factor in this association is likely undernutrition or malnutrition [11].

Among 254 participants, 39 had LBW newborns, and 15 of these LBW newborns were admitted to the NICU. The NICU admission trend was similar to the study by Samsury et al. (2022) [13]. The study exhibited a statistically significant relationship between LBW and NICU admission, corresponding to Inoue et al. (2016) and Haidari et al. (2021) [11,12]. The study also revealed a statistically significant connection between maternal height and NICU admission, corresponding partially to Yearwood et al. (2023), who found that infants born to women of short stature had an increased risk of NICU admission [33].

**Figure 3:**
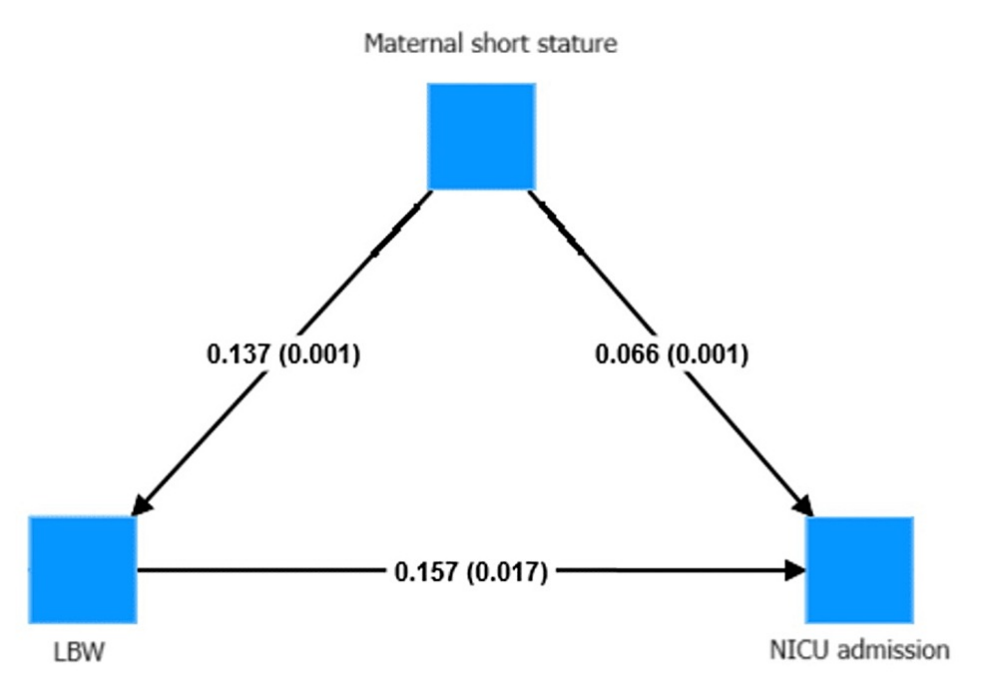
Examining the mediating effect of LBW on NICU admission for maternal short stature LBW: low birth weight, NICU: neonatal intensive care unit

The mediating effect of LBW on NICU admission was assessed to observe whether there was any indirect effect of maternal short stature through LBW (Figure 3). The study revealed no significant mediating effect. The finding implied that LBW did not mediate the association between maternal height and NICU admission. Hence, in contrast to Inoue et al. [11], the link between maternal height and NICU admission was direct.

Our study's strengths include its stratified random sampling, thereby minimizing selection bias. Although the study was conducted only in one tertiary hospital, this is the lone government hospital with a NICU facility in the state, enabling the generalizability of our findings for the entire state. However, there are certain limitations to consider. The study did not specifically examine the impact of undernutrition, which is typically associated with factors such as household income, individual diseases (e.g., gastrointestinal diseases, cancer, and eating disorders), and dietary habits. Again, the study excluded any major medical disorder resulting in LBW babies through a questionnaire; no investigation validated it.

## Conclusions

In conclusion, maternal short stature could be a valuable prenatal indicator for anticipating and planning LBW and NICU admissions. As a screening measure, maternal height may become a valuable tool in assessing these risks in various healthcare settings.
